# Evaluation of post-operative pain after single-sitting pulpectomy using hand versus rotary instrumentation in primary molars

**DOI:** 10.6026/973206300221354

**Published:** 2026-03-31

**Authors:** Anukrati Doneria, Kinjal Shah, Endla Varun Kumar, Devashree Shukla, Neel Gagan, Amish Diwanji, Miral Mehta

**Affiliations:** 1 Department of Dental Surgery, Sarojini Naidu Medical College, Agra, India; 2 Department of Pediatric and preventive dentistry, Manubhai Patel Dental College and Hospital, Vadodara, Gujarat, India; 3 Department of Dentistry, Maharaja's institute of medical sciences, Nellimarla, Vizianagaram, Andhra Pradesh, India; 4 Department of Dentistry, LN Medical College and J K Hospital, Kolar Road, Bhopal, Madhya Pradesh, India; 5 Department of Pediatric and preventive dentistry, Maharaja Ganga Singh Dental College, Sri Ganganagar, Rajasthan, India; 6 Department of Pediatric and Preventive Dentistry, Faculty of Dental Science, Dharmsinh Desai University, Nadiad, India; 7 Department of Pediatric and Preventive Dentistry, Karnavati School of Dentistry, Karnavati University, Gandhinagar, Gujarat, India

**Keywords:** Pulpectomy, primary molars, rotary instrumentation, hand instrumentation, post-operative pain, pediatric dentistry, single-visit endodontics

## Abstract

Post-operative pain after single-sitting pulpectomy in primary molars significantly affects children's treatment acceptance and subsequent behaviour. Therefore, it is of interest to compare hand versus rotary instrumentation in 80 children (4-9 years) requiring pulpectomy in mandibular primary molars (n=40 each). Pain was assessed using the Wong-Baker FACES scale at 6, 12, 24, 48, and 72 hours post-treatment, alongside analgesic consumption. Rotary instrumentation showed significantly lower pain scores at 6 hours (1.85±1.12 vs 3.20± 1.45, p<0.001), 12 hours (1.42±0.98 vs 2.65±1.28, p<0.001), and 24 hours (0.78±0.72 vs 1.55±1.08, p< 0.001). Rotary instrumentation reduces post-operative pain compared to hand files, establishing it as the preferred technique for single- visit pulpectomy in pediatric endodontics.

## Background:

Pulpectomy is a treatment option that is used when there is irreversible pulpitis or necrotic pulp in the primary teeth with a view to eliminating the infection, preserving the tooth until the natural exfoliation and keeping the arch intact to allow the permanent successors to erupt properly [1]. Pulpectomy can be determined by a good biomechanical preparation, good disinfection, and complete obturation of the root canal system. Nevertheless, the secondary tooth root canal (ribbon-like canals) and the lateral canals, together with apical ramifications that compose the complex anatomy of the primary tooth root canals, create considerable technical difficulties when it comes to instrumentation [2]. Root canal preparation in primary teeth has seen the traditional method of conventional hand instrumentation with stainless steel files. Although successful, this method is time- consuming and demands more than one file modification and may contain a high amount of procedural errors, such as ledge creation, canal transfer, and perforation [3]. A long period of treatment is especially a nuisance in pediatric cases, where lack of cooperation and attention span directly affects the quality of treatment and management of the child. NiTi (nickel-titanium) rotary instrumentation systems have transformed the endodontic practice in permanent teeth with the following advantages: less time is required in treatment, better shaping of canals, and better removal of debris [4]. These advantages have led to the consideration of rotary systems of primary tooth pulpectomy. NiTi instruments have a high level of flexibility, so the adapted flexibility allows them to adapt more too curved canals, preserving the original canal curvature, which may help to minimise iatrogenic errors [5]. Endodontic pain after treatment is one of the most frequent patient requests and one of the quality indicators. The level of pain and the length of pain determine patient satisfaction, analgesic needs, and future dental treatment attitudes [6]. Post-operative pain among pediatric patients can affect the behaviour of a patient in further visits and lead to the development of dental anxiety. The knowledge of factors that influence the post-operative pain is therefore necessary in terms of maximising the treatment protocols.

Comparisons of rotary versus hand instrumentation in primary teeth have been made in several studies, most of which have involved clinical and radiographic success rates, instrumentation time, and quality of obturation [7]. Saxena *et al.* conducted a randomized clinical trial in primary molars and reported that although rotary instrumentation significantly reduced instrumentation time, it did not produce a statistically significant reduction in postoperative pain or differences in obturation quality compared with hand files [8]. Yet, there is relatively scarce comparative data that would specifically cover the outcomes of postoperative pain. The process through which the instrumentation technique affects post-operative pain is a multi-factorial process. Apical migration of debris and irrigants, mechanical aggravation of periapical tissues, and the discharge of inflammatory mediators are all reasons for post-procedural discomfort [9]. Theoretically, rotary instrumentation minimises extrusion of the debris due to its crown-down design, which allows its use and constant rotation, which may lead to the reduction of post- operative symptoms [10]. Single-visit pulpectomy has become accepted as a feasible treatment option in treating the primary teeth, with benefits such as fewer appointments, less anxiety regarding the treatment process, and there being no concerns about interappointment leakage [1^11^]. Nonetheless, the instrumentation and obturation process are necessary within the same visit, and it is required to be performed with efficient methods so that the time spent on it is minimal and the preparation of the canal is sufficient. Rotary instrumentation incorporated into single-visit protocols could maximise the outcomes of efficiency, as well as patient comfort. Although there is increased literature on rotary instrumentation in primary teeth, there is a major research gap on systematic assessment of post-operative pain following single-sitting pulpectomy in comparison of hand and rotary instruments. Instrumentation time and clinical success have been described in most of the existing studies, whereas pain outcomes are either examined as secondary outcomes or not assessed at all [12]. Moreover, pain assessment protocols designed to be used with children have not been used systematically. Therefore, it is of interest to compare post-operative pain after single-sitting pulpectomy with the use of conventional hand instrumentation and rotary instrumentation in the primary mandibular molars using validated pediatric pain assessment scales and standardised follow-up regimens.

## Materials and Methods:

## Study design and setting:

This prospective, parallel-group, randomised controlled trial was conducted at the Department of Pediatric and Preventive Dentistry between March 2022 and November 2023.

## Sample size calculation:

Sample size was calculated based on pilot study data and previous literature reporting mean pain score differences of 1.5 units (SD=2.0) between instrumentation techniques at 24 hours post-operatively. With α=0.05, power=80%, and allocation ratio of 1:1, a minimum of 36 subjects per group was required. Considering a 10% dropout rate, 40 subjects were enrolled in each group, totalling 80 participants.

## Participant selection:

## Inclusion criteria:

[1] Healthy children aged 4-9 years (ASA I classification)

[2] Mandibular primary first or second molar requiring pulpectomy

[3] Clinical diagnosis of irreversible pulpitis or pulp necrosis

[4] Restorable tooth with sufficient crown structure

[5] At least two-thirds root length remaining radiographically

[6] Cooperative behaviour (Frankl rating 3 or 4)

[7] Parent/guardian consent and child assent

## Exclusion criteria:

[1] Systemic diseases affecting pain perception or healing

[2] History of analgesic or antibiotic use within 7 days

[3] Presence of periapical abscess with extra-oral swelling

[4] Internal or external root resorption exceeding one-third root length

[5] Perforation of the pulp chamber floor

[6] Previously initiated endodontic treatment

[7] Known allergy to local anaesthetics or study materials

[8] Teeth with anatomical anomalies

[9] Uncooperative behaviour during treatment

## Randomisation and allocation:

Eligible participants were randomly allocated to either the hand instrumentation group (Group H) or the rotary instrumentation group (Group R) using computer-generated random numbers. Allocation concealment was maintained using sequentially numbered, opaque, sealed envelopes opened only after patient enrollment and anaesthesia administration. Due to the nature of the intervention, operator blinding was not possible; however, outcome assessors and statisticians were blinded to group allocation.

## Pre-operative assessment:

All patients underwent comprehensive clinical and radiographic examination before treatment.

Pre-operative parameters recorded included:

[1] Demographic information (age, gender)

[2] Tooth involved (first vs. second molar)

[3] Pulpal diagnosis (irreversible pulpitis vs. necrosis)

[4] Presence of pre-operative pain

[5] Pre-operative periapical status (normal vs. radiolucency)

[6] Baseline anxiety level (Modified Child Dental Anxiety Scale)

## Treatment protocol:

All the pulpectomy procedures were conducted by one calibrated pediatric dentist who had five years of experience in the two methods of instrumentation. Treatments were done in one session, utilising a standardised procedure.

## Anaesthesia and access:

The 2 per cent lignocaine with 1:80,000 adrenalines was used to administer an inferior alveolar nerve block. The isolation was done with a rubber dam using suitable clamps. An access cavity was prepared with high-speed round burs with water coolant, and carious tissue was excavated. Extirpation of coronal pulp tissue with a sharp spoon excavator, the pulp chamber roof was removed totally.

## Determination of working length:

Its radiographic working length was set at 1-2mm below the radiographic apex with an electronic apex locator (Propex Pixi, Dentsply Sirona) and verified radiographically. Every canal was measured separately.

## Hands and instrumentation (Group H):

Laboratory instrumentation of root canals was done through stainless steel K-files (Mani Inc., Japan) in a step-back technique. Canals were first negotiated with #15 K-file, then successively until a canal anatomy of choice (#30 or 35) was reached. It was filmed in push-pull motion and quarter-turn rotation. Between the file sizes, irrigation of 1% sodium hypochlorite (10 mL total per canal) was done with a side- vented needle 2 mm below the working length. Ending irrigation was done with 17% EDTA (2 mL) and a saline rinse.

## Rotary instrumentation (Group R):

The instrumentation of root canals was carried out by use of Kedo-S pediatric rotary file system (Reeganz Dental Care Pvt. Ltd., India), having three files (U1: 25/0.08 taper; E1: 25/0.06 taper; E2: 25/0.04 taper). The sequence was through files and brushing with 300 rpm and 2.2 Ncm torque with X-Smart Plus endomotor (Dentsply Sirona). Group H Irrigation protocol, which was the same as Group H, was carried out between files.

## Canal drying and obturation:

Sterile paper points were used to dry the canals. The process of obturation was undertaken with zinc oxide eugenol cement blended to a creamy consistency. Lentulo spiral was used at a slow speed to introduce cement, after which the master cone was added where necessary. Unnecessary cement was eliminated, and the access cavity was covered with glass ionomer cement (GC Fuji IX, GC Corporation, Japan).

## Post-operative instructions:

Parents were given standard verbal and written instructions about diet, oral hygiene and pain management. Ibuprofen suspension (10 mg/kg) was prescribed to be used as a rescue analgesic for moderate-severe pain only. Analgesic administration was to be recorded by parents.

## Outcome assessment:

## Primary outcome - postoperative pain:

Wong-Baker FACES Pain Rating Scale (0-10) was used to measure pain, which is a valid scale when working with children. An evaluation was done at 6, 12, 24, 48, and 72 hours after the operation. Training of the parents on scale administration was done during the appointment, and the parents were contacted by telephone at designated intervals.

## Secondary outcomes:

[1] Analgesic use (dose number of 72 hours)

[2] Incidences of swelling (parent-reported)

[3] Sleep disturbance (yes/no)

[4] Normal functioning recovery (hours after treatment)

[5] Unscheduled emergency cases.

## Time recording instrumentation:

The time of a total instrument was counted from the initial file insertion to the end of the final irrigation time, counted by a digital stopwatch. This was to eliminate the preparation of access, working length and obturation time.

## Statistical analysis:

The SPSS version 25.0 (IBM Corporation, Armonk, NY) was used to analyse the data. The continuous variables were given as the mean ± standard deviation, and the categorical variables were given as frequencies and percentages. The Shapiro-Wilk test was used to test the normality. Independent samples t-test or Mann-Whitney U test, depending on the appropriate use, were used to obtain between-group comparisons about pain scores. Repeated-measure ANOVA was used to measure changes in pain scores with time. Categorical variables were compared using chi-square test and Fisher's exact test. Cohen's d was used to obtain the effect size. A p-value less than 0.05 were taken to be statistically significant.

## Results:

All 80 enrolled participants completed the study with no dropouts. Both groups were comparable regarding demographic characteristics, tooth distribution, pulpal diagnosis, and pre-operative parameters. The mean age was 6.2 ± 1.4 years in Group H and 6.4 ± 1.3 years in Group R (p=0.492). Baseline characteristics are presented in Table 1 (see PDF). Mean instrumentation time was significantly shorter in Group R compared to Group H (8.6 ± 2.4 minutes vs. 16.8 ± 4.2 minutes; p<0.001). The reduction in instrumentation time with the rotary technique was 48.8%. Group R demonstrated significantly lower pain scores compared to Group H at 6, 12, and 24 hours post- operatively. At 48 and 72 hours, pain scores remained lower in Group R, but differences were not statistically significant. Detailed pain score comparisons are presented in Table 2 (see PDF). Repeated measures ANOVA revealed significant effects of time (F=186.4; p<0.001), group (F=42.8; p<0.001), and time x group interaction (F=8.6; p<0.001) on pain scores, indicating differential pain trajectories between groups. Pain severity was categorised as no pain (score 0), mild (score 2), moderate (score 4-6), and severe (score 8-10). At 6 hours, moderate-severe pain was reported by 35.0% in Group H compared to 12.5% in Group R (p=0.016). At 24 hours, 72.5% of Group R patients reported no pain compared to 45.0% in Group H (p=0.012). Analgesic consumption was significantly lower in Group R compared to Group H. Sleep disturbance and time to normal activity resumption also favoured the rotary group. Secondary outcome comparisons are presented in Table 3 (see PDF). Subgroup analysis revealed that children with pre-operative pain demonstrated greater benefit from rotary instrumentation (pain score difference at 6 hours: 1.68 vs. 0.72 in patients without pre-operative pain; interaction p=0.024). No significant interactions were observed for age group, tooth type, or pulpal diagnosis. Two patients in Group H required emergency visits within 72 hours for persistent moderate pain; both were managed with additional analgesics without the need for retreatment. No patients in Group R required emergency intervention. No instrument separation, perforation, or other procedural complications occurred in either group.

## Discussion:

This randomised control trial indicates that pulpectomy performed with a single sitting with rotary instrumentation produces a significantly lower level of post-operative pain than the conventional hand instrumentation on primary mandibular molars. The rotary technique was clinically significant in the reduction of pain during the first 24 hours post-treatment period, and also with less need for analgesics and better secondary effects such as quality sleep and restoration of activity. The difference in the mean pain score of 1.35 units after 6 hours is clinically significant since a difference above 1.0 unit on the Wong-Baker FACES scale has been found to be significant in children [13]. The high effect sizes (Cohen d >0.8) of the findings at initial time points also provide further evidence as to the clinical relevance of the study results. The duration of pain relief was 24 hours, which was the maximum length of the post-operative pain, which allows most patients to complain about during the endodontic procedures. The process involved in the lowering of post-surgical pains with rotary instrumentation is possibly the lessening of apical debris extrusion. Rotary systems have the inherent crown-down technique, which eliminates coronal debris first, then proceeds in an apical direction, reducing the piston effect that drives infected material through the apex [14]. Comparisons made between extrusions of the rubbish when using instrumentation methods invariably showed the extrusion of the rubbish using rotary instrumentation as lower in volume than extrusion using hand instrumentation in primary teeth. There is also a shorter instrumentation time when using rotary (48.8 time cut in our experiment), which is translated into a lower mechanical irritation of periapical tissues. The long-term manipulation of the root canal system provokes an enhanced inflammatory response, and it promotes the emerging post-operation symptoms [15]. Rotary instrumentation efficiency reduces the trauma caused by the procedure and still ensures sufficient quality of preparation. In our instrumentation time results, we find agreement with the reports of other studies that have indicated a 40-60% reduction in time of instrumentation using a rotary in comparison to the time of instrumentation using hands in primary teeth. This efficiency in time not only has the benefit of decreasing the post-operative pain but also increases the child's behaviour in treatment [16]. Increased fatigue is reduced by shorter appointments, cooperation is preserved, and quality treatment is achieved before attention runs away, especially in young, nervous patients. A major patient-centred outcome is the much lower analgesic consumption of the rotary group (22.5 vs. 47.5% needed analgesics). Minimised medication demand decreases possible negative effects, eases the care following the surgery among parents, and demonstrates real enhanced comfort with reduced pain instead of hypnotised pain [17]. The uniformity in the rescue analgesic protocol meant that groups had similar access to pain management. Sleep disturbance after dental treatment occurs in both children and their families, and it may have implications on the functioning of the next day and attitude towards dentistry. The minimal difference in sleep disturbance among rotary (15.0% vs. 35.0) indicates that minimised post-operative pain corresponds to the better outcome of quality of life [18]. Likewise, a more rapid recovery to normal activities means fewer effects on the day-to-day functioning.

The observation that children who had undergone pre-operative pain were found to receive better treatment because of rotary instrumentation indicates some differences in pre-operative periapical tissue sensitivity or inflammatory conditions. Painful teeth might have more reactive periapical tissues, and therefore softer instrumentation practices may be especially beneficial [19]. This observation should be investigated in more detail in subgroup analyses that are adequate. Lack of instrumental separation in our case indicates that the technique was correctly applied, and the characteristics of the design of the rotary systems are pediatric. The Kedo-S system employed in this paper has reduced working length and adjustable NiTi fabrication that is tailored to the anatomies of primary teeth [20]. The training of operators and following the manufacturer's instructions in terms of speed, torque, and pecking motion adds to the procedural safety. Parent satisfaction scores were more favourable in the rotary population, as they indicated the combination of the advantages of less time of therapy, less distress of the child during the procedure, and better post- operative outcome. Patient/parent satisfaction is a quality indicator that carries significant implications for treatment acceptance and dental attendance patterns [21]. The increased satisfaction could lead to better cooperation in the further restorative visits. Methodological differences have to be considered to compare it with the existing literature. The past studies, which investigated the post-operative pain after primary tooth pulpectomy, have used different assessment instruments, timing and definition of the outcomes [22]. Internal validity is enhanced through our use of validated pediatric pain scales, standardised intervals of assessment and blinded outcome assessment. The single-operator design removed the inter-operator variation but can restrain the generalizability. The instrumentation technique was made the variable of interest by the standardised irrigation protocol using the same volumes and concentrations in both groups. The effect of irrigation itself is that it causes post-operative results due to its tissue dissolution and the effect of removing debris [23]. The future study may involve the synergetic effects of optimised irrigation procedures and rotary instrumentation. Here are a number of restrictions that should be mentioned. The design is single-center which might be a limitation of external validity. Although parent-reported pain assessment is required by practical considerations, it creates the possibility of reporter bias. The follow-up period was relatively limited to discuss the immediate post-operative pain; the use of prolonged clinical and radiographic outcomes would have been better considered separately. Also, the fact that only mandibular molars are used does not allow generalising to the results of maxillary teeth, where anatomic variations can play a role. The clinical outcome of this research justifies the use of rotary instrumentation to use in preference over single sitting pulpectomy in primary molars. The overall advantages of decreased post-operative pain, decreased treatment time, less analgesic needs, and enhanced parent satisfaction give sufficient reasons leading to the adoption of the rotary technique [24]. Training activities ought to focus on pediatric dental practitioner rotary instrumentation competence.

## Conclusion:

We show rotary instrumentation for single-sitting pulpectomy significantly reduces post-operative pain (48.8% less at 24 hours), instrumentation time, and analgesic intake versus hand files in primary mandibular molars. Rotary methods improve sleep quality, accelerate activity recovery, and increase parent satisfaction beyond pain reduction alone. Rotary instrumentation should become the preferred pediatric endodontic technique, with future studies needed for long-term success, maxillary applications, and optimal file systems.
